# Frequency of circulating topoisomerase-I-specific CD4 T cells predicts presence and progression of interstitial lung disease in scleroderma

**DOI:** 10.1186/s13075-016-0993-2

**Published:** 2016-05-04

**Authors:** Andrea Fava, Raffaello Cimbro, Fredrick M. Wigley, Qing-Rong Liu, Antony Rosen, Francesco Boin

**Affiliations:** Department of Medicine, Division of Rheumatology, Johns Hopkins University School of Medicine, 5200 Eastern Avenue, MFL Building, Center Tower, Suite 4100, Baltimore, MD 21224 USA; Behavioral Neuroscience Research Branch, National Institute of Drug Abuse, National Institutes of Health, 251 Bayview Boulevard, Baltimore, Maryland 21224 USA; Department of Medicine, Division of Rheumatology, University of California, San Francisco, 513 Parnassus Avenue, Med Sci, S-847, San Francisco, CA 94143 USA

**Keywords:** Systemic sclerosis, Interstitial lung disease, T lymphocytes, Th17, Autoantigen

## Abstract

**Background:**

Scleroderma is an antigen-driven T cell-mediated autoimmune disease. Presence of anti-topoisomerase-I antibodies is associated with pulmonary fibrosis and predicts increased mortality. Characterization of autoreactive T lymphocytes may shed light on disease pathogenesis and serve as a biomarker for disease activity. Here, we aimed to quantify and functionally characterize circulating topoisomerase I (topo-I)-specific CD4+ T cells and to define their association with presence and progression of interstitial lung disease (ILD) in patients with scleroderma.

**Methods:**

Using flow cytometry, circulating topo-I-reactive CD4+ T cells were identified by the expression of specific activation markers (CD154 and CD69) upon stimulation with purified topo-I and quantified in 27 SSc patients and 4 healthy donors (HD). Polarization of autoreactive T cells (Th1, Th2, Th17, Th1–17) was defined using surface expression of specific chemokine receptors. Presence and progression of ILD were determined using high-resolution chest CT and pulmonary function tests.

**Results:**

Topo-I-reactive CD4+ T cells were found in all topo-I-positive patients compared to one topo-I-negative subject and no HD. Topo-I-specific CD4+ T cells exhibited a distinct Th17 polarized phenotype. Autoreactive T cells were significantly increased in subjects with evidence of ILD and were quantitatively associated with the decline of lung volumes.

**Conclusions:**

Topo-I-specific T cells can be reliably quantified in the peripheral blood of patients with scleroderma, exhibit a pro-inflammatory Th17 phenotype, and predict progression of ILD.

**Electronic supplementary material:**

The online version of this article (doi:10.1186/s13075-016-0993-2) contains supplementary material, which is available to authorized users.

## Background

Systemic sclerosis (SSc) is characterized by obliterative microangiopathy, progressive fibrosis of affected organs, and autoimmunity [1]. Early in the disease, an inflammatory mononuclear cell infiltrate precedes fibrosis and parallels the development of vasculopathy in affected tissues, suggesting that a disrupted immune response is a major driver in SSc pathogenesis [2]. A more precise understanding of the immune effector pathways would allow identifying reliable markers to monitor disease activity and to define suitable targets for a selective therapeutic intervention.

In scleroderma, the presence of specific autoantibodies is strongly associated with distinct clinical phenotypes [3]. In particular, anti-topoisomerase-I (topo-I) antibodies have high specificity for scleroderma, and are present in 20–45 % of patients [4, 5]. They are associated with diffuse skin involvement and pulmonary fibrosis, and independently predict more severe disease and mortality [6, 7]. Despite these robust associations, the serum anti-topo-I antibody levels are not useful to monitor disease activity and progression in SSc patients [8–10], and there are no convincing data supporting a direct role for these autoantibodies in the pathogenesis of SSc suggesting that the anti-topo-I response is involved but not antibody mediated.

Importantly, anti-topo-I antibodies undergo class switching and exhibit a strong association with human leukocyte antigen (HLA) alleles across different ethnic groups, implicating T cells in this response [11, 12]. The presence of an antigen-driven, T cell-dependent process in SSc is supported by several studies [12–16]. Defining the functional phenotype of topo-I-reactive T cells would be relevant to determine whether and how this cellular subset may contribute to inflammation and tissue damage in SSc. However, no conclusive data have been provided to define whether autoantigen-specific T cell responses are amplified in individuals with active disease and contribute to the generation of distinct phenotypes through their effector function. These aspects have been difficult to investigate due to poor accessibility of target tissues and technical challenges to detect ex vivo low-frequency antigen-specific T cells [12–16]. In this study, we employed a novel assay to identify, quantify and functionally characterize topo-I-specific CD4+ T cells. This is based on the detection upon antigen stimulation of reliable markers of early activation (i.e., CD154, CD69) as well as of T cell polarization. We found that topo-I-responsive T cells are selectively increased in anti-topo-I-positive SSc patients and exhibit a T helper (Th)17 pro-inflammatory functional phenotype, with frequency quantitatively associated with presence and progression of interstitial lung disease (ILD). These findings implicate amplified topo-I-specific T cell responses in the pathogenesis of SSc lung disease, and suggest that they may be appropriate targets to monitor disease activity and predict outcome.

## Methods

Patients evaluated at the Johns Hopkins Scleroderma Center were included in the study after providing written informed consent if they met the American College of Rheumatology preliminary criteria for the classification of SSc [17]. The Johns Hopkins institutional review board approved the study.

### Clinical phenotyping

Demographic and clinical data including age, sex, ethnicity, smoking status, disease duration, scleroderma subtype, specific organ involvement, use of immunosuppressive agents, and autoantibody status were obtained at the time of the visit. Severity of skin involvement was quantified using the modified Rodnan skin score [18]. Pulmonary involvement was determined based on abnormal pulmonary function tests (PFT), including measurements of forced vital capacity (FVC) and single-breath carbon monoxide diffusing capacity (DL_CO_), calculated according to the American Thoracic Society recommendations [19]. All PFT data were standardized to same reference values [20, 21]. Presence of ILD was confirmed by the detection of established fibrotic lung changes on computerized tomography of the chest. ILD progression was defined as % change in FVC in the year preceding the test date or in the subsequent 10 months in those patients for whom follow-up data became available. Lung disease severity was assessed using the Medsger severity scale [22].

### T cell culture and stimulation

Peripheral blood mononuclear cells (PBMC) were isolated from whole blood through density-gradient centrifugation (Ficoll-Paque Plus, GE Healthcare, Chicago, IL, USA), resuspended in RPMI medium supplemented with 5 % autologous serum, 2 mM L-glutamine, 100 U/ml penicillin, and 100 ug/ml streptomycin, transferred onto a 96-well plate (1.5 × 10^6^ cells/well) and cultured at 37 °C. To prevent CD154 internalization, PBMCs were preincubated with anti-human CD40 blocking antibody (1 ug/ml; G28.5, BioLegend, San Diego, CA, USA) for 15 minutes. Subsequently, cells were stimulated for 18 hours using alternatively 1 ug/ml staphylococcal enterotoxin B (SEB)/staphylococcal enterotoxin A (SEA), 15 ug/ml tetanus toxoid (TT) (NIBSC), 17 ug/ml human recombinant topoisomerase-I purified from baculovirus-infected insect cells (GenScript Biotech Corp, Piscataway, NJ, USA), 18 ug/ml purified human recombinant (baculovirus) protein arginine methyltransferase 6 (PRMT6) (Cayman Chemicals, Ann Arbor, MI, USA) or human peptidyl arginine deiminase type 4 (PAD4) (gift from Dr. Erika Darrah).

### Flow cytometry

After stimulation and washings with PBS, cells were then stained with Live/Dead Fixable Blue Dead Cell Stain Kit (Molecular Probes, Eugene, OR, USA), BV510-conjugated anti-CD3 (OKT3, BioLegend), Pacific Blue-conjugated anti-CD4 (RPA-T4, BD Pharmingen Franklin Lakes, NJ, USA), APC-H7-conjugated anti-CD8 (SK1, BD, Franklin Lakes, NJ, USA), PE-conjugated anti-CD154 (TRAP1, BD Pharmingen), APC-conjugated anti-CD69 (FN50, BD Pharmingen), Alexa-fluor 488-conjugated anti-CXCR3 (TG1/CXCR3, BioLegend), PerCP-Cy5.5-conjugated anti-CCR6 (TG7/CCR6, BioLegend), PE-Cy7-conjugated anti-CCR4 (1G1, BD Pharmingen) antibodies, and flow cytometry acquisition was performed on a FACSAria (BD) instrument. Analysis was conducted using FlowJo software (Tree Star Inc., Ashland, OR, USA).

### Cytokine quantitation

Freshly isolated PBMCs were sorted according to the surface expression of the chemokine receptors CCR6, CXCR3, and CCR4 (Fig. 3a), resuspended in RPMI medium supplemented with 10 % fetal bovine serum (FBS), 2 mM L-glutamine, 100 U/ml penicillin and 100 ug/ml streptomycin, and then stimulated with anti-CD3/CD28 beads (Life Technologies, Carlsbad, CA, USA) at a bead-to-cell ratio of 2:1 for 48 hours (37 °C). Supernatants were collected and stored at -80 °C. Quantitation of interferon (IFN)γ, interleukin (IL)-4 and IL-17A was performed in duplicate using DuoSet-ELISA kits (R&D Systems, Minneapolis, MN, USA) according to manufacturer’s instructions.

### RNA isolation and cDNA synthesis

Topo-I specific CD4+ T cells identified after 18 h stimulation as described in the Methods section of the main manuscript were sorted according to their chemokine receptor expression pattern into 50 μl of RNA extraction buffer (PicoPure RNA isolation kit, Arcturus Bioscience, Inc., Mountain View, CA, USA), incubated at 42 °C for 30 min and centrifuged (800 × g) at RT for 2 min. The supernatant was then collected in an RNase-free tube. Subsequent column filtration, washing, and elution of RNA from the columns were performed according to section C of the PicoPure RNA isolation protocol (Protocol for Use with CapSure Macro Laser Capture Microscope Caps). RNA integrity numbers were measured using Agilent RNA 600 Pico kit (Agilent Technologies, Santa Clara, CA, USA). Single-strand cDNAs were synthesized with the Superscript III first-strand cDNA synthesis kit following the manufacturer’s protocol (Invitrogen, Life Technologies, Waltham, MA, USA).

### Pre-amplification and quantitative real-time polymerase chain reaction (RT-qPCR)

TaqMan PreAmp Master Mix Kit was used for cDNA preamplification (Applied Biosystems, Life Technologies, Waltham, MA, USA) using pooled primer mixes (200 times dilution) of the predesigned TaqMan assays of IL17A (Hs00174383_m1), IL4 (Hs00174122_m1), IFNG (Hs00989291_m1) and endogenous control beta-actin (4326315E). cDNAs were preamplified in an ABI 9700 Thermal Cycler using the program: 95 °C hold for 10 min, and then 14 cycles of denaturation at 90 °C for 15 sec and annealing and extension at 60 °C for 4 min. The pre-amplification PCR products were immediately diluted five times with molecular biology-grade water (5 PRIME, Gaithersbrug, MD, USA) and stored at -20 °C or immediately processed for qPCR. Duplex qPCR assays were performed on technical duplicates using a Fam-labeled probe for each target gene and a Vic-labeled probe for the endogenous control gene, along with TaqMan® Advanced Fast PCR Master Mix (Life Technology). RT-qPCR reactions were run in a 7500 Fast TaqMan instrument using the program: 95 °C hold for 20 sec followed by 40 cycles of denaturation at 95 °C for 3 sec and annealing and extension at 60 °C for 30 sec. Calculations of relative expression from Ct data were carried out according to User Bulletin #2 for ABI Prism 7900 Sequence Detection System.

### Enzyme-linked immunosorbent assay (ELISA)

Anti-topoisomerase-I antibody concentration was measured in the serum of SSc patients using QUANTA Lite Scl70 enzyme-linked immunosorbent assay (Inova Diagnostics, San Diego, CA, USA) according to the manufacturer’s instructions.

### Statistical analysis

Statistical analysis was performed using Prism 6.0 Software for Macintosh (GraphPad Software, San Diego, CA, USA). Differences between variables were determined using Student *t* test and Wilcoxon rank-sum test for continuous variables, and the χ^2^ or Fisher’s exact test for categorical variables. Multiple comparisons were performed by analysis of variance (ANOVA) with Bonferroni correction for normally distributed variables or Kruskal-Wallis and Dunn’s test otherwise. Linear associations were analyzed using Pearson correlation coefficient upon assessment for Gaussian distribution with Shapiro-Wilk test. Throughout, a two-tailed α of 0.05 was used.

## Results

We studied 27 consecutive SSc patients: 15 anti-topo-I-positive and 12 anti-topo-I-negative. The two groups were similar with regards to the main demographic and disease characteristics (Table 1). Consistent with previous studies, anti-topo-I positive patients exhibited a significant higher prevalence of ILD (*p* = 0.005), worse modified Rodnan Skin Score, and a trend for lower diffusion capacity of lung for carbon monoxide (DLco).

**Table 1:** Patient demographics and clinical characteristics

Variables	SSc Patients	Topo-I negative (N = 12)	Topo-I positive (N = 15)	*P* value^§^
Age (years)^*^	51.3 ± 9.6	55.1 ± 7.4	48.2 ± 10.3	.093
Female	21 (78)	9 (75)	12 (80)	.557
Race				
White	22 (81)	9 (75)	13 (87)	.338
Black	5 (19)	3 (25)	2 (13)	
Smoking status				
Never	16 (62)	9 (82)	7 (47)	.174
Past	1 (4)	0 (0)	1 (7)	
Current	9 (34)	2 (18)	7 (47)	
Diffuse SSc skin type	15 (56)	5 (42)	10 (67)	.182
mRSS^*^ (range 0–51)	7 ± 8	5 ± 9	9 ± 7	.037
SSc duration				
RP onset^*^, years	11.1 ± 7.8	11.2 ± 7.3	10.9 ± 8.5	.516
1st non-RP symptom^*^, years	10.2 ± 7.1	11.0 ± 7.2	9.6 ± 7.3	.792
Lung severity score^*^ (range 0–4)	1.2 ± 1.3	0.9 ± 1.5	1.5 ± 1.1	.168
FVC^*^ (% predicted)	77.9 ± 14.7	81.4 ± 19.8	77.07 ± 11.1	.106
DLCO^*^ (% predicted)	75.3 ± 16.6	83.8 ± 15.7	71.3 ± 17.3	.051
ILD^†^	11 (46)	1 (10)	10 (71)	.005
eRVSP^*^	28.8 ± 12.5	32.5 ± 17.5	25.9 ± 5.9	.522
Autoantibody status				
ACA	3 (11)	3 (25)	0 (0)	.075
Anti-RNA-polymerase III	5 (21)	3 (25)	1 (8)	.295
Immunosuppression (current)^‡^	11 (41)	4 (33)	7 (47)	.381

All values are given as number (%) unless otherwise specified. Frequencies (%) are calculated based on available data. SSc disease duration was calculated at the time of serum sampling from the onset of RP or from the first non-RP symptom. Lung severity score is reported as previously defined by Medsger et al. [29]

*ACA* anticentromere antibody, *DLCO*, diffusion capacity of lung for carbon monoxide, *eRVSP* right ventricular systolic pressure estimated by echocardiography, *FVC* forced vital capacity, *ILD* interstitial restrictive lung disease, *mRSS* modified Rodnan skin score, *RP* Raynaud’s phenomenon, *SSc* systemic sclerosis

^*^Mean ± SD

^†^The presence of ILD was defined by presence of fibrosis on computed tomography of the chest (CT chest was available in 10 topo-I-negative and 14 topo-I-positive subjects)

^‡^Use of immunosuppressants includes cyclophosphamide, mycophenolate, methotrexate, or prednisone

^§^ *P* values were determined by Fisher’s exact test, Pearson chi-square, or the Wilcoxon rank-sum test, as appropriate

### CD154 and CD69 upregulation identifies topoisomerase-I-reactive CD4+ T cells

**Fig. 1:**
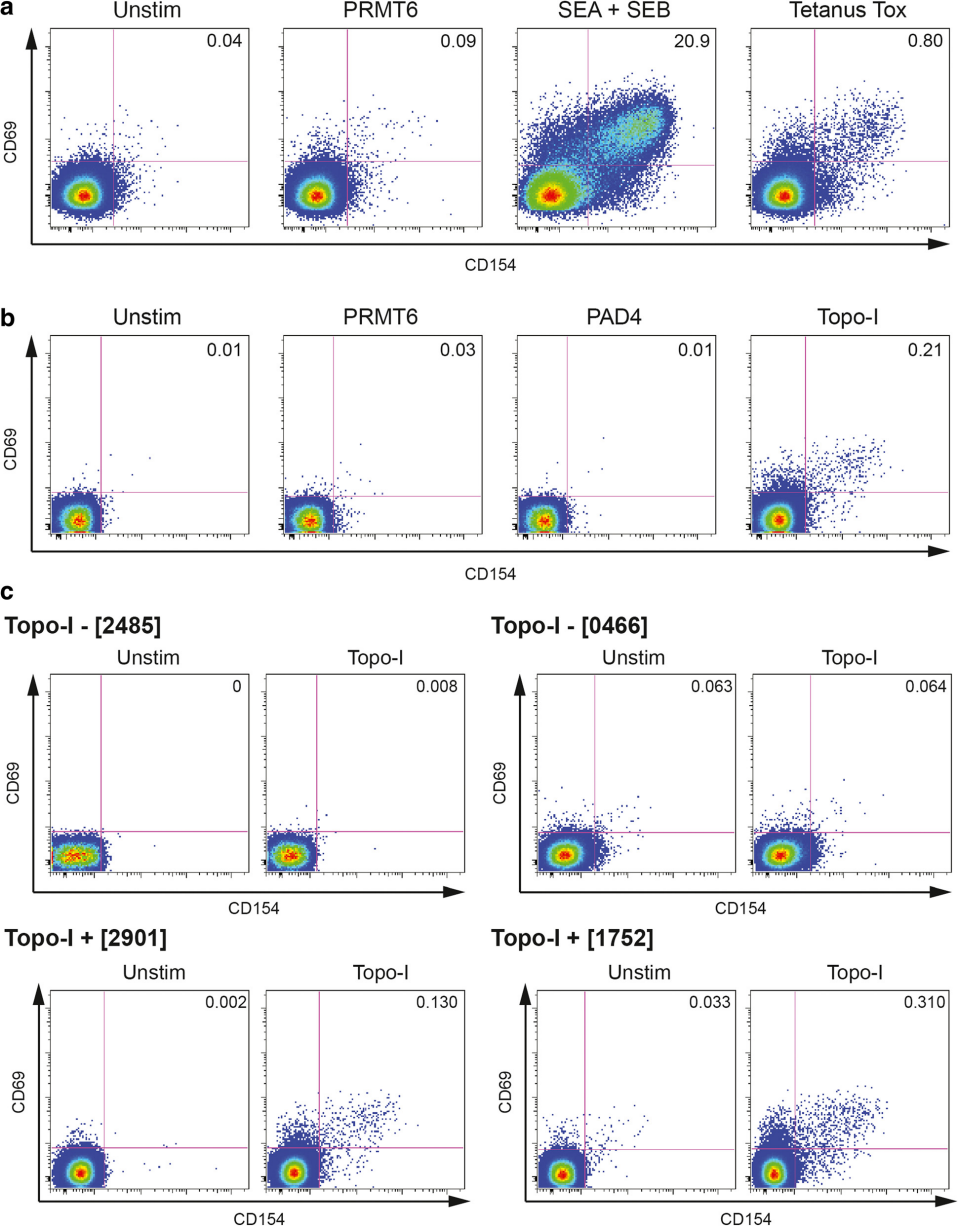
Detection of circulating topoisomerase-I-specific CD4+ T cells by CD154 and CD69 expression and upregulation. After incubation (15 min) with anti-CD40 blocking antibodies, freshly isolated peripheral blood mononuclear cells (PBMCs) were stimulated for 18 h in presence of 5 % autologous serum as indicated below. Gating was set on lymphocytes, singlets, live, CD3+ and CD4+ cells. Numbers in quadrants indicate the percent (%) cells in the parent CD4+ population. **a** PBMCs from a healthy donor recently immunized with combined tetanus, diphtheria, and pertussis (Tdap) vaccine were stimulated with PRMT6 (negative control), SEA + SEB (positive control) and tetanus toxoid (TT). **b** PBMCs from a topo-I+ SSc patient were stimulated with PRMT6, PAD4 (negative controls), or topo-I. **c** Representative experiments on two anti-topo-I-negative and two anti-topo-I-positive SSc patients. The gating strategy for this experiment is shown in Additional file 1: Figure S1. *PRMT6* protein arginine methyltransferase 6, *PAD4* human peptidyl arginine deiminase type 4

In order to detect with reliability and specificity antigen-specific T cells, we developed a flow cytometry-based protocol measuring the expression of early activation markers CD69 and CD154 on CD4+ T cells. First, we optimized the assay using PBMCs obtained from a healthy donor recently immunized with combined tetanus, diphtheria, and pertussis (Tdap) vaccine. After stimulation for 18 hours with tetanus toxoid (TT) in the presence of anti-CD40 blocking antibodies and 5 % autologous serum, a selective upregulation of both CD69 and CD154 was detected on CD4+ T cells (0.8 %), while no significant response was observed in unstimulated cells (0.04 %) or after exposure to a negative control protein (0.09 %) (Fig. 1a). The gating strategy for this experiment is shown in Additional file 1: Figure S1. This approach was then applied to SSc patients using the whole topo-I molecule purified from baculovirus-infected insect cells and two unrelated proteins for which no T cell autoreactivity was expected in SSc (PRMT6 and PAD4) as negative controls. A representative experiment is shown in Fig. 1b. Topo-I-reactive CD154 + CD69 + CD4+ T cells were specifically identified in topo-I-positive SSc patients, while no response was triggered by either PRMT6 or PAD4.

### Topoisomerase-I-specific CD4+ T cells are increased in anti-topo-I-positive SSc patients

**Fig. 2:**
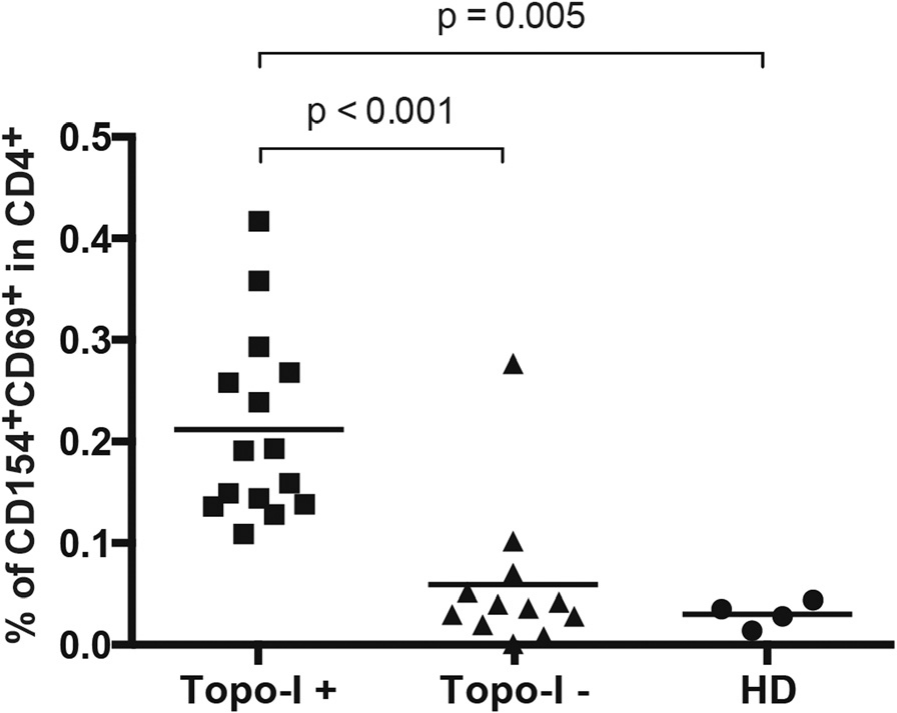
Increased frequencies of topoisomerase-I-specific CD4+ T cells in the blood of anti-topo-I-positive SSc patients. Topo-I-specific CD4+ T cells frequency was measured in the peripheral blood of 15 anti-topo-I-positive, 12 anti-topo-I-negative SSc patients, and 4 healthy donors (HD). Values represent the percentage of CD154 + CD69+ cells within the CD4+ population. To minimize any noise from the background, the frequency of topo-I-reactive T cells was calculated after subtracting the small percentage of CD154 + CD69 + CD4+ T cells detected within unstimulated PBMCs from the same subject. *Horizontal lines* indicate the mean frequency for each group

Next, we sought to define the magnitude of autoreactive CD4+ T cell expansion in the peripheral blood of SSc patients. CD4+ T cells from anti-topo-I-positive subjects consistently exhibited a significant upregulation of CD69 and CD154 upon topo-I challenge compared to unstimulated cells and those from anti-topo-I-negative patients (Fig. 1c). Autoreactive CD154 + CD69 + CD4+ topo-I-specific T cells were found in all 15 topo-I-positive patients compared to only one topo-I-negative subject and no healthy donors (HD), with a frequency ranging between 0.136 % and 0.417 % of total CD4+ T cells (topo-I-positive vs topo-I-negative *p* < 0.001; topo-I-positive vs HD *p* = 0.005; topo-I-negative vs HD *p* = ns) (Fig. 2).

### Recognition of topoisomerase-I by autoreactive CD4+ T cells is HLA-DR restricted

**Fig. 3:**
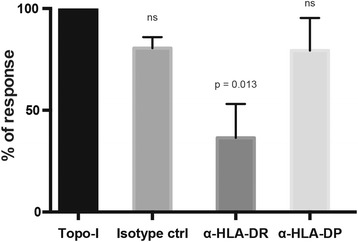
Topoisomerase-I-specific CD4+ T cells activation is HLA-DR restricted. PBMC from topo-I-positive SSc patients were cultured for 18 h with topo-I and anti-HLA-DR or anti-HLA-DP blocking antibodies or isotype control (1 ug/ml). Values are represented as percent of response relative to the frequency of CD154 + CD69 + CD4+ cells detected after stimulation with topo-I alone as assessed by flow cytometry. Data are representative of three separate experiments (mean ± SD)

As CD4 T cells recognize antigens in the context of major histocompatibility complex (MHC) class II molecules, we asked whether T cell response to topo-I was MHC-restricted and in particular mediated by HLA-DR alleles [23]. PBMCs from topo-I-positive patients were stimulated with topo-I in presence of anti-HLA-DR or -HLA-DP blocking antibodies. Positive responses to topo-I were significantly decreased by HLA-DR blockade (*p* = 0.013), while anti-HLA-DP antibodies did not impact T cell activation (Fig. 3), implicating that specific topo-I epitopes presented to the T cell receptor (TCR) by HLA-DR molecules can selectively induce and maintain autoreactive T helper immune responses.

### Topoisomerase-I-specific CD4+ T cells exhibit a Th17 polarized phenotype

**Fig. 4:**
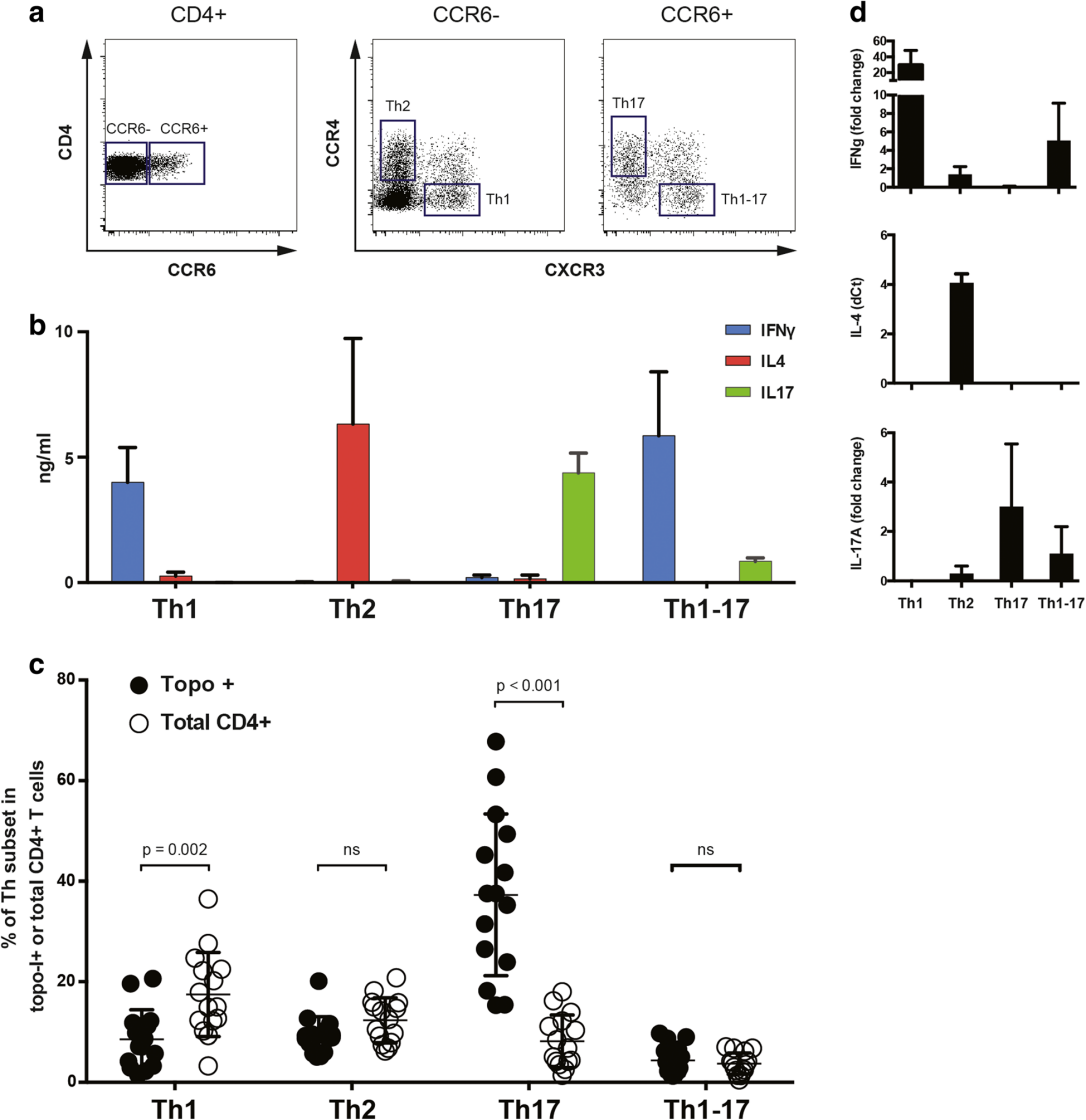
Topoisomerase-I-specific CD4+ T cells show a Th17 polarized functional phenotype. **a** CD4+ T cell subsets were identified by flow cytometry using the combined surface expression of chemokine receptors CCR6, CXCR3, and CCR4. **b** After sorting, the different T helper subsets were stimulated with anti-CD3/CD28 beads for 48 h and secretion of IFNγ, IL-4, and IL-17A was measured in their supernatants by enzyme-linked immunosorbent assay (*bars* represent mean ± SE, n = 3). **c** The distribution of polarized T helper subsets within topo-I-specific CD4+ T cells (*closed circles*) is shown in comparison to the general CD4+ population (*open circles*). *T* test with Bonferroni correction was used for multiple comparisons. Lines indicate mean ± SD. **d** After sorting topo-I-specific CD4+ T cells according to their polarized T helper phenotype (range 16–2064 cells), mRNA expression levels of IFNγ, IL-4, and IL-17A were measured by qPCR using the Arcturus® PicoPure® RNA Isolation system, which is designed to recover high-quality RNA from a very low number of cells (10–500 cells) and to accomplish cDNA synthesis from minimal amounts of cDNA. IFNγ and IL-17A expression was calculated as fold change in reference to a mix of topo-I-reactive CD4+ T cells from three patients; IL-4 expression is reported as delta Ct from actin expression since it was not detectable in the reference population. Data are representative of four separate patients (mean ± SE)

To characterize the functional properties of topo-I-specific CD4+ T cells, we validated and employed an approach to identify polarized T helper subsets based on the surface expression of specific chemokine receptors [24]. Th1, Th2, Th17 and Th1-Th17 CD4+ T cells were defined by different combinations of CXCR3, CCR6 and CCR4 expression (Fig. 4a). After cell sorting and stimulation of each individual subset with anti-CD3/CD28 beads, the production of specific cytokines was assessed in the supernatants, confirming their polarized status (Fig. 4b). CCR6 + CCR4 + CXCR3- T cells (Th17) selectively secreted IL-17A, CCR6 + CCR4-CXCR3+ T cells (Th1–17) both IFNγ and IL-17A, CCR6-CCR4 + CXCR3- T cells (Th2) exclusively IL-4 and CCR6-CCR4-CXCR3+ T cells (Th1) mainly IFNγ. Based on these findings, we found that CD4+ T cells responding to topo-I stimulation, exhibited a distinct enrichment in the Th17 subset (*p* < 0.001) compared to the total CD4+ population, while their Th1 polarization was decreased (*p* = 0.002) (Fig. 4c). The frequency of Th2 and Th1–17 subsets did not differ between topo-I-specific and total CD4+ T cells. To further test the specificity of these phenotypes, IFNγ, IL-4 and IL-17A mRNA expression was quantified in topo-I-responsive T cells sorted according to their polarized status. The cytokine specificity of autoreactive polarized subsets was confirmed at the transcriptional level (Fig. 4d). In particular, topo-I-specific CCR6 + CCR4 + CXCR3-CD4+ T cells selectively expressed IL-17A mRNA.

### Topoisomerase-I specific CD4+ T cells are quantitatively associated with presence and progression of ILD

**Fig. 5:**
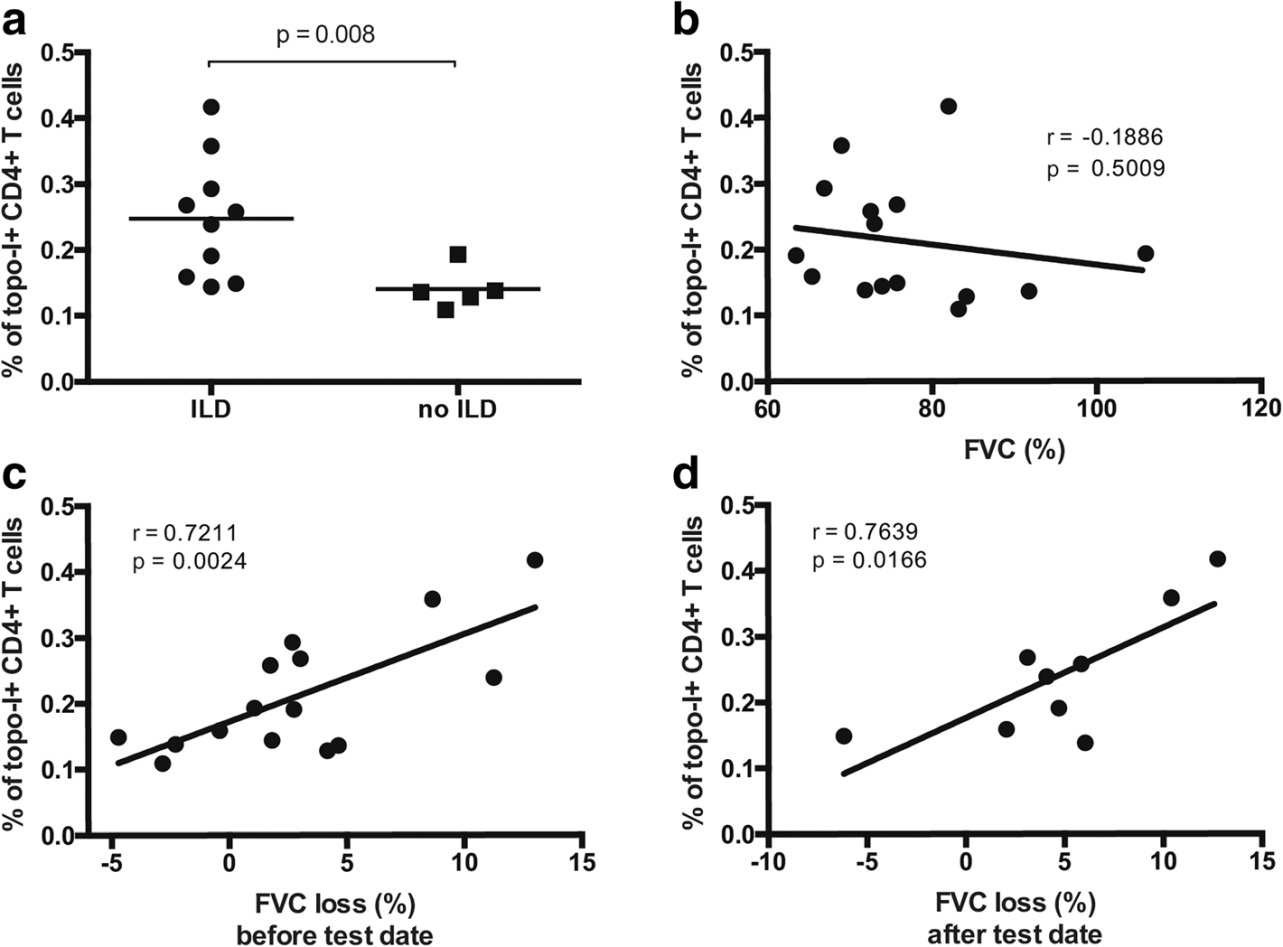
Frequency of topoisomerase-I-specific CD4+ T cells is associated with presence and progression of interstitial lung disease (ILD). **a** Frequency of topo-I reactive CD4+ T cells in anti-topo-I-positive SSc patients stratified by presence of ILD. *Lines* indicate means. *P* value was calculated using Wilcoxon rank-sum test. **b** Association of topo-I-specific CD4+ T cells with FVC (% predicted). **c** and **d** Associations between topo-I-specific CD4+ T cells frequency and the degree of ILD progression defined as % change in forced vital capacity (FVC) in the year preceding the test date (**c**) or in the subsequent 10 months (**d**) in those patients for whom follow-up data became available. Pearson correlation coefficient r and *p* values are displayed in **b**, **c**, and **d**

**Fig. 6:**
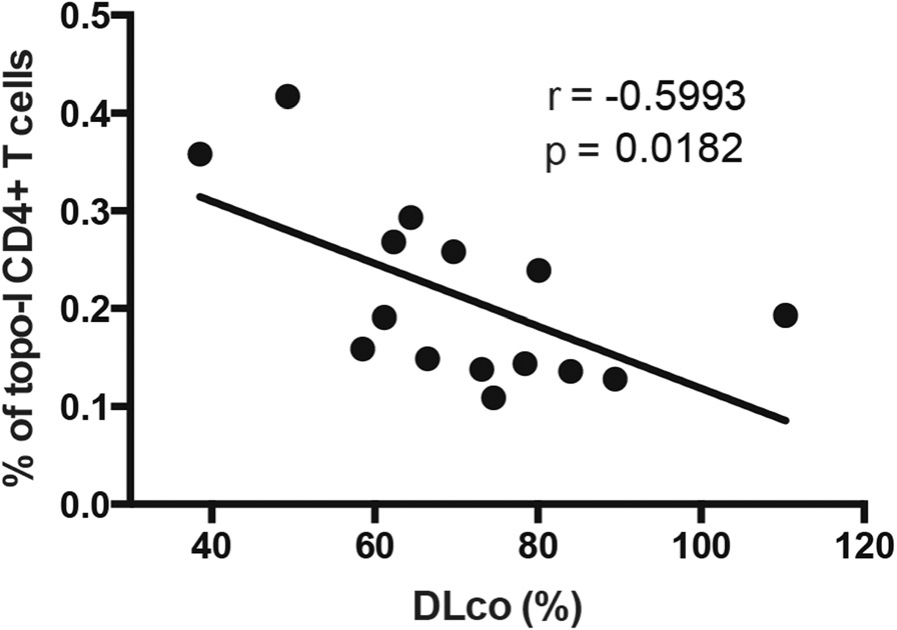
Frequency of topoisomerase-I-specific CD4+ T cells exhibits negative association with the diffusion capacity of lung for carbon monoxide (DLco). Association of topo-I-specific CD4+ T cells with DLco (% predicted) in SSc patients. Pearson correlation coefficient r and *p* values are displayed

**Fig. 7:**
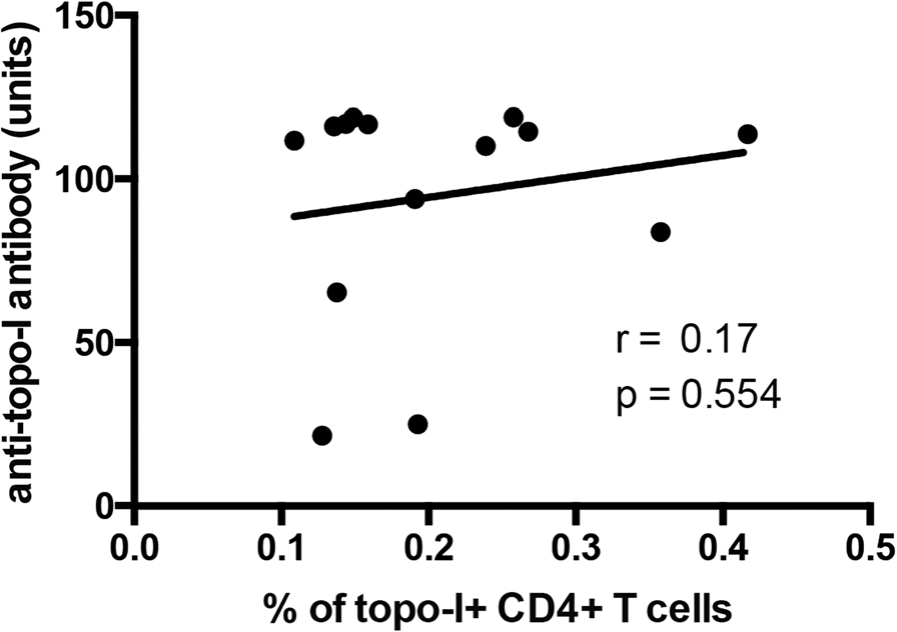
Frequency of topoisomerase-I-specific CD4+ T cells does not correlate with anti-topoisomerase-I antibody serum concentration. Serum anti-topoisomerase-I antibody concentration is measured in units of reactivity compared to a standard low positive sample as per manufacturer’s instructions. Pearson correlation coefficient r and *p* value are displayed

**Fig. 8:**
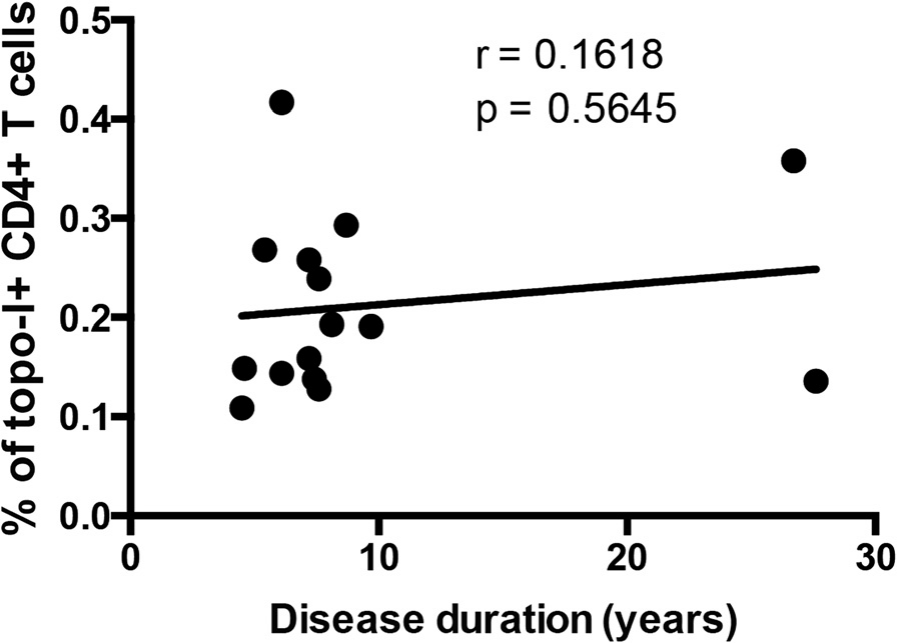
Frequency of topoisomerase-I-specific CD4+ T cells is not associated with disease duration. Disease duration is calculated in years from the first non-Raynaud’s phenomenon symptoms to the time of blood sampling. Pearson correlation coefficient r and *p* values are displayed

Several studies have confirmed that anti-topo-I positivity is significantly more prevalent among SSc patients with pulmonary fibrosis and entails worse clinical outcome. Therefore, we next addressed these important clinical associations and sought to define whether the frequency of topo-I-specific T cell responses might have clinical utility. Topo-I-reactive T cells were significantly increased in the peripheral blood of patients with ILD compared to those without lung involvement (*p* = 0.002) (Fig. 5a). A lower DLco was associated with higher number of topo-I-specific T cells (Pearson r = -0.6, *p* = 0.018) (Fig. 6) but no association was found with the FVC (Fig. 5b). In contrast, the proportion of circulating topo-I-specific CD4+ T cells was quantitatively associated with the progression of lung fibrosis as defined by the loss of lung volumes within the year preceding the test date (Pearson r = 0.721, *p* = 0.002) (Fig. 5c) or during the subsequent one (Pearson r = 0.764, *p* = 0.017) (Fig. 5d). None of these clinical associations were found with anti-topo-I serum titers (Additional file 2: Figure S2). Importantly, the frequency of topo-I-specific T cells did not show any linear relationship with anti-topo-I titer or with disease duration (Figs. 7 and 8).These data suggest that autoreactive T cell activation against topo-I is sustained during progression of SSc-ILD and predicts further loss of lung volumes.

## Discussion

The evidence that a distinct immune-mediated process may drive target tissue injury in scleroderma has not been paralleled by the ability to define with precision its magnitude and the functional features of involved cellular and molecular effectors. In this study, we have developed a reliable method to identify and quantify topoisomerase-I-specific CD4+ T cells with high specificity and precision, determining that in SSc patients topo-I-specific T cells are strongly polarized toward a pro-inflammatory Th17 phenotype and quantitatively associated with the presence and progression of ILD.

Lung fibrosis is the most common pulmonary manifestation in SSc and a leading cause of morbidity and mortality [25, 26]. A substantial group of SSc-ILD patients (15–25 %) progress towards end-stage lung disease [27]. Regrettably, treatment options for ILD have been limited to nonselective immunosuppression. Therapeutic efficacy has been hindered by our poor ability to diagnose lung involvement earlier, to effectively monitor the disease course and to identify highly selective pathogenetic targets. Currently, the only reliable tool to predict disease activity and progression in SSc-ILD is the prospective measurement of lung volumes, as high-resolution chest computed tomography or bronchoalveolar lavage fluid analysis have shown lack of sensitivity and/or specificity both before or after therapeutic intervention [28, 29]. Importantly, the fact that ILD progression is often identified by spirometry criteria only after irreversible lung damage has already occurred remains one of the major obstacles to the prompt initiation of therapy and ultimately to its efficacy. The findings of this study indicate that quantification and functional profiling of topo-I-specific CD4+ T cells may represent a novel, useful, and noninvasive tool to assess and predict disease activity in SSc-ILD. Moreover, the quantitative association of these autoreactive T cells with the degree of progressive FVC decline suggests that expansion of the topo-I autoreactive T cells and ongoing lung injury may be mechanistically connected.

The identification of circulating autoreactive CD4+ T cells is challenging due to their very low frequency. Previous studies pursuing the detection of topo-I-specific T cells in SSc patients have yielded limited results due to relatively weak cellular responses to topo-I as well as poor specificity, as autoreactive T cells have been found also in anti-topo-I-negative patients as well as healthy controls [12–14, 16]. Most of these investigations used bulk assays measuring proliferation of mononuclear cells by ^3^H-thymidine incorporation after several days of in vitro stimulation and could not reliably determine the frequency of circulating topo-I-specific T cells nor provide insight about their functional phenotype. In addition, these studies did not yield any data regarding the association between topo-I-reactive T cells and relevant clinical outcomes. To overcome these problems, we used full-length topo-I obtained from a baculovirus-insect cell expression system and adopted a method that relies on markers of antigen-driven T cell activation such as CD69 and CD154 [30, 31]. CD154 upregulation is highly specific for T cell receptor (TCR) engagement with cognate antigens and is not influenced by nonspecific stimuli such as activating cytokines, providing our study with the specificity and sensitivity needed to detect rare cellular events [31]. Additionally, CD154 is expressed primarily on CD4+ T cells within few hours, making it the ideal marker to probe early activation events in response to autoantigen stimulation [31]. Such early activation is typical of antigen-experienced cells, which are uniquely and reliably identified by our method. This provides a clear advantage over other bulk assays where a significant background noise may be generated by nonspecific activation of naïve T cells in response to the culture conditions or topo-I. In fact, subjects with shared HLA-DR alleles associated with anti-topo-I antibody positivity may have low-level T cell clone proliferation in response to the autoantigen [12, 32].

The presence of topo-I specific CD4+ T cells is remarkably restricted in our study to patients with anti-topo-I antibodies. As lung fibrosis may occur in topo-I-negative patients, other antigens, known (i.e., U1 RNP, U3 RNP) or yet unknown, may be targeted and drive the disease process. Our experimental approach can be applied to study these other autoantigens as well as to investigate other rheumatic conditions where the number and functional properties of autoreactive T cells targeting disease-specific autoantigens may be mechanistically related to the underlying fibrotic lung disease (i.e., antisynthetase syndrome).

Anti-topo-I antibodies are class switched, indicating that topo-I-specific T cells play a significant role in B cell activation and autoantibody production. However, while anti-topo-I antibody positivity is strongly associated with development of SSc-ILD and independently predicts mortality, serum levels do not correlate reliably with disease activity and progression [5–10]. This further suggests that T cells may contribute to the pathogenesis of the disease through their direct pro-inflammatory and immunomodulatory effect.

We found that CD4 T cell responses to topo-I are HLA-DR restricted, which confirms and extends previous reports indicating that anti-topo-I positivity is significantly associated with HLA-DRB1*1104 alleles [23]. This finding should further support pursuing the identification of allele-specific HLA-DR-binding topo-I peptides and the development of tools such as class II peptide-MHC complexes tetramers, which can enhance our ability to monitor autoantigen-specific T cell responses in blood and within target tissues.

Our study shows robustly that topo-I-specific CD4+ T cells exhibit a chemokine receptor and cytokine expression profile indicative of a Th17 polarized phenotype. While the frequency of Th17 lymphocytes is low in the peripheral blood of healthy individuals, this subset may undergo recruitment and expansion in sites of active inflammation and tissue damage, as shown in several autoimmune disorders [33–35]. The tight association between autoreactive T cells and the declining lung function detected in our SSc-ILD patients suggests a possible mechanistic link between selective Th17 activation and progression of lung damage. In animal models, IL-17 can drive fibrosis, as shown in the lungs and skin of bleomycin as well as tight-skin-1 (TSK-1/+) mice [36, 37]. In addition, immunization of C57BL/6 mice with recombinant human topo-I and Freund’s complete adjuvant promotes the development of SSc-like manifestations in association with increased production of IL-6 and IL-17 as well as higher levels of circulating IL-17-secreting T cells (Th17) [38]. IL-17 can also activate TGF-β and CTGC pathways in fibroblasts derived from mouse skin, and promote collagen production after epithelial-mesenchymal transition in mouse alveolar epithelial cells [37]. In contrast, a direct Th17-mediated pro-fibrotic function in humans has not been proven yet. Nonetheless, human fibroblasts express functional IL-17 receptors and respond to IL-17 stimulation with activation and proliferation [39]. Skin biopsies obtained from SSc patients have shown increased expression of collagen mRNA in fibroblasts nearby inflammatory infiltrates and higher numbers of IL-17+ T cells located in tight proximity of myofibroblasts, implying a cross-talk between these two cell types [40]. Therefore, Th17 T cell activation may indirectly contribute to the fibrotic process in SSc by driving early local inflammatory events and amplifying the magnitude of fibroblast response to other direct pro-fibrotic stimuli present in affected tissues. In fact, increased levels of IL-17 and Th17 cells have been found in the peripheral blood as well as within target tissues of SSc patients during early disease and in association with ILD [41, 42]. Cytokines crucial for Th17 T cell priming and expansion such as IL-6 and IL-23 have been reported enriched in SSc subjects [43, 44]. IL-6 secretion by topo I-specific T cell clones has shown to be crucial for effective activation and production of anti-topo-I antibodies by autologous B cells obtained by SSc patients [45]. In addition, polymorphism of the IL-23 receptor gene (*IL23R*) has shown association with diffuse SSc and anti-topo-I positivity [46]. In our previous work, we have shown that an increased frequency of circulating Th2/Tc2 polarized T cells in SSc patients is significantly associated with the presence of pulmonary fibrosis [47]. Other studies have detected a predominance of T cells producing IFNγ (Th1) both in the periphery and within affected organs during earlier phases of the disease, with subsequent decline during late-fibrotic stages [48]. This emphasizes that the participation of T cells in SSc pathogenesis may not be exclusively limited to a direct pro-fibrotic function and varies in terms of intensity and phenotype during the course of the disease.

## Conclusions

Our investigation shows that quantification and functional profiling of autoantigen-driven immune responses can be employed to predict with precision the presence and progression of underlying fibrotic lung disease in SSc. Prospective studies will determine whether assessing topo-I-specific T cell responses is useful to measure more precisely the efficacy of therapeutic interventions during routine clinical care and in experimental trials. More readily, this approach can be applied to investigate the presence and the function of autoreactive T cells within target organs, providing important insight about tissue-specific pathways and mechanisms of injury relevant for SSc onset and progression.

### Ethics approval and consent to participate

Patients evaluated at the Johns Hopkins Scleroderma Center were included in the study after providing written informed. The Johns Hopkins institutional review board approved the study.

## Additional files

Additional file 1: Figure S1. Flow cytometry gating strategy for CD4+ T cells. G1: live lymphocytes identified in peripheral blood mononuclear cells (PBMCs) according to the forward and sideward scatters (FSC-A/SSC-A). G2, G3: singlet selection conducted on FSC-H/FSC-W and SSC-H/SSC-W dot plots to avoid cellular doublets. G4: dead cells exclusion (Live/Dead Fixable Blue Dead Cell Stain Kit, Molecular Probes). G5, G6: gating on CD3+ and CD4+ T lymphocytes. Numbers indicate percentage of parent population. (TIF 12577 kb) Additional file 2: Figure S2. Serum levels of anti-topo-I antibodies are not associated with severity and progression of ILD. (A and B) Association of anti-topo-I antibodies serum concentration with FVC (% predicted) and DLco (% predicted). (C and D) Associations between anti-topo-I antibodies serum levels and degree of ILD progression defined as % change in forced vital capacity (FVC) in the year preceding the test date (C) and in the subsequent 10 months (D). Pearson correlation coefficient r and *p* values are displayed. (TIF 12770 kb)

## Abbreviations

ANOVA: analysis of variance
DLco: diffusion capacity of lung for carbon monoxide
ELISA: enzyme-linked immunosorbent assay
FBS: fetal bovine serum
FVC: forced vital capacity
HD: healthy donor
HLA: human leukocyte antigen
IFN: interferon
IL: interleukin
ILD: interstitial lung disease
MHC: major histocompatibility complex
PAD4: protein arginine deiminase 4
PBMC: peripheral blood mononuclear cells
PCR: polymerase chain reaction
PFT: pulmonary function test
PRMT6: protein arginine methyltransferase 6
SSc: systemic sclerosis
TCR: T cell receptor
Th: T helper
topo-I: anti-topoisomerase-I
TT: tetanus toxoid
