# Supplementary figures and images for: Frequency of circulating topoisomerase-I-specific CD4 T cells predicts presence and progression of interstitial lung disease in scleroderma
Source: Arthritis Res Ther. 2016 May 4;18:99. doi: 10.1186/s13075-016-0993-2 (PMC4857293; doi:10.1186/s13075-016-0993-2)

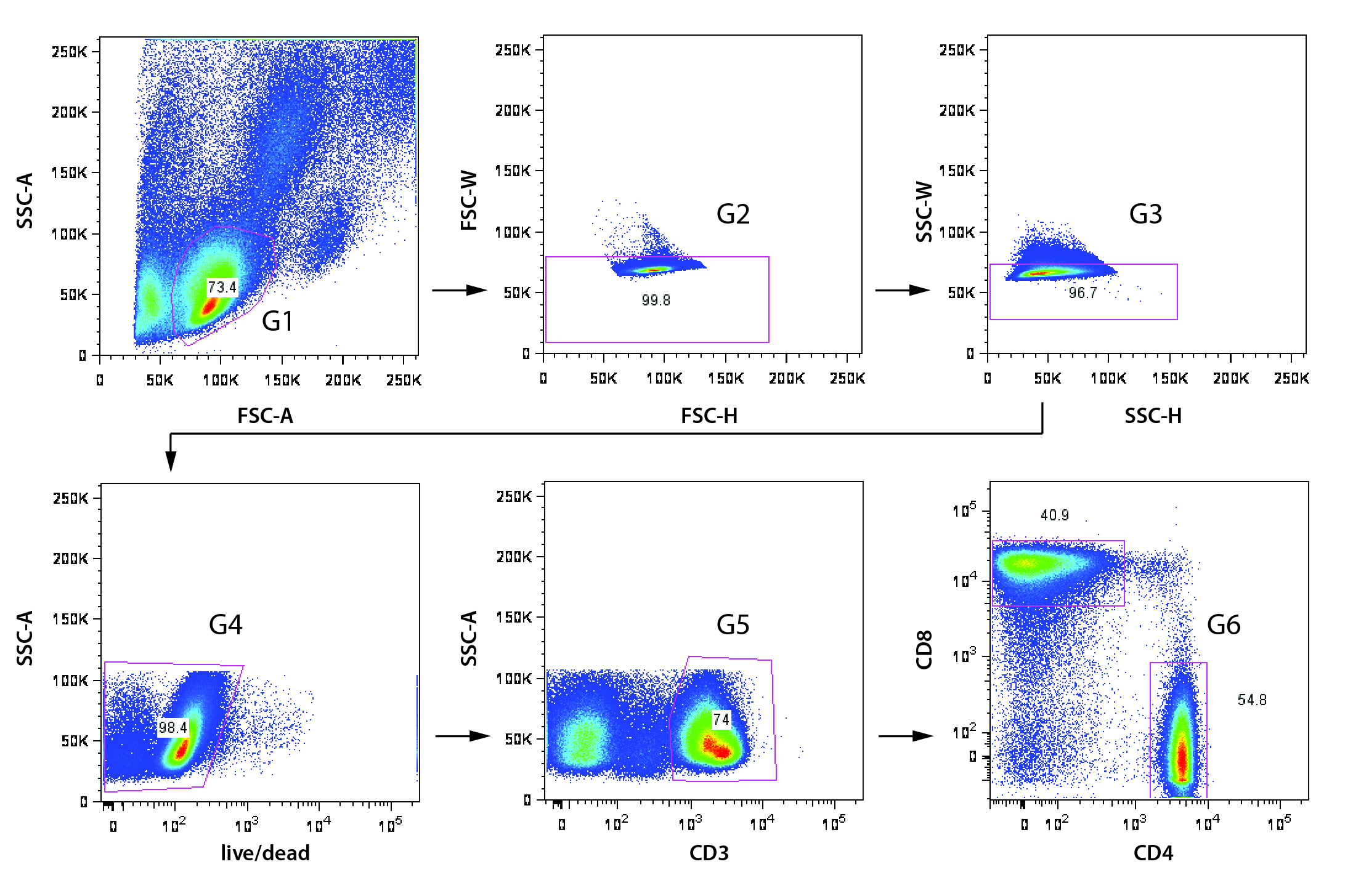

Supplement: Additional file 1: Figure S1. — Flow cytometry gating strategy for CD4+ T cells. G1: live lymphocytes identified in peripheral blood mononuclear cells (PBMCs) according to the forward and sideward scatters (FSC-A/SSC-A). G2, G3: singlet selection conducted on FSC-H/FSC-W and SSC-H/SSC-W dot plots to avoid cellular doublets. G4: dead cells exclusion (Live/Dead Fixable Blue Dead Cell Stain Kit, Molecular Probes). G5, G6: gating on CD3+ and CD4+ T lymphocytes. Numbers indicate percentage of parent population. (TIF 12577 kb) [file 13075_2016_993_MOESM1_ESM.tif]

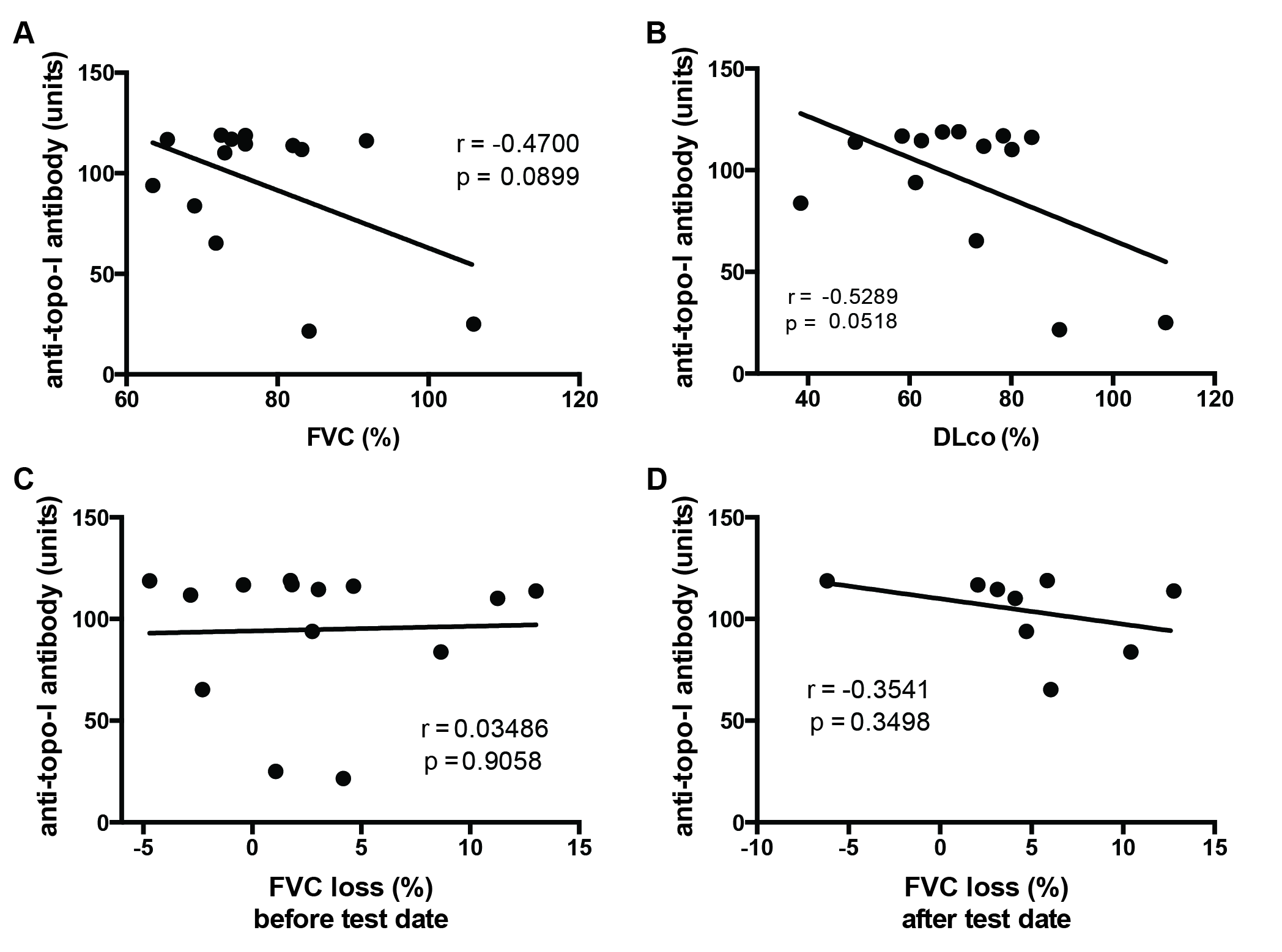

Supplement: Additional file 2: Figure S2. — Serum levels of anti-topo-I antibodies are not associated with severity and progression of ILD. (A and B) Association of anti-topo-I antibodies serum concentration with FVC (% predicted) and DLco (% predicted). (C and D) Associations between anti-topo-I antibodies serum levels and degree of ILD progression defined as % change in forced vital capacity (FVC) in the year preceding the test date (C) and in the subsequent 10 months (D). Pearson correlation coefficient r and p values are displayed. (TIF 12770 kb) [file 13075_2016_993_MOESM2_ESM.tif]
